# Clinical and demographic characteristics of secluded and mechanically restrained mentally ill patients: a retrospective study

**DOI:** 10.1186/s13584-018-0274-4

**Published:** 2019-02-01

**Authors:** Chanoch Miodownik, Michael D. Friger, Eyal Orev, Yisroel Gansburg, Nadav Reis, Vladimir Lerner

**Affiliations:** 1 Be’er Sheva Mental Health Center, Faculty of Health Sciences, Ben-Gurion University of the Negev, Box 4600, 84170 Be’er-Sheva, PO Israel; 2 Department of Public Health, Faculty of Health Sciences, Ben-Gurion University of the Negev, Be’er Sheva, Israel

**Keywords:** Inpatients, Psychiatric diagnosis, Restraint, Seclusion

## Abstract

**Background:**

Restraint or seclusion measures in acute psychiatric care are used as a last resort when all other methods for removal of physical threat have failed. The purpose of this study is to find a correlation between coercive measures, demographic characteristics within this patient group, and factors associated with shortened periods of restriction.

**Methods:**

This is a one-year retrospective study conducted in a male acute closed ward of a psychiatric hospital in Israel. The data from January 1, 2014 to December 31, 2014 were retrieved from the records of patients who underwent restraint and/or seclusion interventions during this period. The analyzed data included age, psychiatric diagnosis, marital status, education, race, ethnicity, length of hospital stay, legal status during admission, type of coercive measure (mechanical restraint, seclusion), number and duration of coercive episodes, reasons for coercion, time of event, number of previous hospitalizations, aggression in past and present treatment, and treatment during events.

**Results:**

During this time period, there were 563 admissions in the study ward. Over this period, 176 subjects (31.3%) underwent 488 restraints and/or seclusions. 98% were aggressive in the past. (Although some results reached statistical significance, we prefer to emphasize here only the most important results, while the others will be presented in the text.) Patients with personality disorders were physically limited for the longest time, while schizophrenia patients were restricted for the shortest time compared with other diagnoses (*p* = 0.007). A negative correlation was found between the length of coercion and the number of academic female nurses on duty (*p* = 0.005), as well as the administration of sedative medications during the restricting procedure.

**Conclusions:**

We believe that the presence of registered, academic female nurses on duty and medication administration during coercive measures can reduce the length of restriction.

## Background

Every country has different laws regarding mentally disturbed patients who need involuntary medical intervention and its own system to cope with them. The public mental health administration in Israel is managed by district psychiatrists, who have the legal authority to force hospitalization and/or force treatment after a voluntary or forced psychiatric examination.

Since 1991, in accordance with the Israeli law for treating mentally ill persons, there have been three categories for involuntary hospitalizations: a) by a district psychiatrist, b) by court for forensic observation, and c) by court for forced treatment.

The problem of violent behavior in psychiatric facilities remains very relevant in the clinician’s everyday practice. Despite significant progress in the pharmacological treatment of mental disorders, mechanical restraint and seclusion are still used in daily practice for psychiatric inpatients. Professional staff usually prefers to avoid using these procedures, since they limit a patient’s freedom and impair the patient’s dignity. However, sometimes it is necessary to do so in order to contain extreme episodes of dangerous behavior threatening the patient’s surrounding environment. In general, restraint is needed for immediate control of physically threatening and/or agitated behavior. This behavior usually resolves and does not recur following the patient’s release [1, 2].

The correct use of coercive measures in a psychiatric ward is a major challenge. It is essential to ensure that these interventions are used only when other approaches have failed. Most of the studies in this field were published over the first decade of the twenty-first century; and according to a recent review, restraint use remains frequent [3]. About 6–17% of psychiatric inpatients undergo this intervention and its prevalence may reach as high as 38% in some involuntarily admitted psychiatric patients [3, 4]. For various reasons, about 24% of all patients admitted to a psychiatric emergency department require restraint or a combination of seclusion and restraint [5]. Published data concerning coercive measures use in various countries are presented in Table 1.

**Table 1:** Data about Restrained and Secluded Inpatients from the Literature

Author (Ref.*)	Country	Number of patients	Study design	% restr.*	% secl.*	Duration of restr.* (h*)	Duration secl.* (h*)	Duration hosp.* (d*)	N previous hosp.*
Allen & Currier [36]	USA	No data	1 year study	71(abs* No.)	308 (abs* No.)	3.2	3.6	No data	No data
Betemps et al. [37]	USA	86 medical centers	1-year study	No data	–	Mean 25.6 M = 16.2	–	No data	No data
Chien et al. [19]	Hong Kong	30	qualitative exploratory study	–	–	Range 2–20 mean 8	–	No data	No data
Dumais et al. [20]	Canada	2721	2-year retrospective	17.5	23.2	Mean 22.4 (SD = 66.6)	Mean 134.3 (SD = 682.4)	Mean 118.1 (SD = 201.0)	No data
Kaltiala-Heino et al. [8]	Finland	1543	6-months retrospective study	3.8	6.6	No data	No data	No data	No data
Kaltiala-Heino et al. [5]	Finland	1543	retrospective chart review	7.2	23.9	Mean 19.4 (SD = 21.9)	Mean 35.8 (SD =76.8)	No data	No data
Keski-Valkama et al. [38]	Finland	1990–6417 1991–6103 1994–5785 1998–5170 2004–4859	Comparative structured postal survey from the national register	1.2 0.7 1.5 0.8 0.8	1.5 1.8 1.2 1.1 1.3	M = 6.7 M = 5.4 M = 6.7 M = 7.3 M = 7.0	M = 5.6 M = 9.0 M = 11.2 M = 12.1 M = 17.1	No data	No data
Korkeila et al. [27]	Finland	1543	6-months retrospective chart review	3.8	6.6	Cumulative 86	Cumulative 574	No data	No data
Kostecka & Zardecka [39]	Poland	414	Comparative study	15.7	–	Mean 7.95 (SD = 4)	–	No data	No data
LeGris et al. [9]	Canada	85	8-months	–	48.2	–	8.7 days	Mean 41 (SD = 31)	Mean 4.2 (SD = 6)
Martin et al. [40]	Germany	31,399	1-year study in 7 German and	49.9	50.1	Mean 9.6 (M = 9.3)	Mean 7.4 (M = 9.0)	No data	No data
	Switzerland	7931	7 Swiss psych hospitalization	20.7	79.3	Mean 48.7 (M = 52.8)	Mean 55.0 (M = 55.1)		
Odawara et al. [41]	Japan	1334	4- years retrospect study	18	–	Within 24 h or less 16.2% Within 2–3 days 24% Within 4–7 days 21.6% Within 8–14 days 14.1% Within 15–30 days 17.9% > 31 days 6.2%	–	Mean 51.4 (SD = 48.2)	2.2
Porat et al. [42]	Israel	1419	A month study in 9 government psychiatric hospitalization	14.2	–	5.4	–	No data	0–26 1–3- 23 4–9 – 21 10+ − 19
Thomas et al. [12]	UK	193	2-years admission cohort	–	44	–	No data	No data	Mean 3.08 (SD = 5.03)
Tunde-Ayinmode & Little [11]	Australia	253	1-year retrospective study	–	31	–	Mean 9 (SD = 10)	Mean 29 (SD = 22)	71% had prev. Hosp.

*Abs* – absolute number, *hosp* – hospitalization Ref – references, *restr* – restraint, secl – seclusion, *M* – median;

*SD* – standard deviation, *h* – hours, *d* - days

A literature review and survey of international trends in psychiatric hospitals in developed countries found great variations in the incidence of seclusion, from 3.6 to 15.6%, and in the incidence of restraint, 1.2 to 8.0% [6]. However, several other studies have reported a range in the incidence of seclusion without restraint in adult psychiatric settings between 4 and 44% [7], and a range in use of seclusion with restraints between 4% [8] and 12% [9]. Longer hospital stay has been associated with a greater risk of seclusion with or without restraint [10, 11]. Studies have also found that patients who have been secluded stay longer in the hospital [9–11], and they have had more previous admissions [12]. In recent decades, psychiatric staff has tried intentionally to reduce the use of coercion, in accordance with international professional ethical guidelines [13].

The present study investigates characteristics of adult inpatients in a closed male psychiatric ward in Israel who underwent restraint and/or seclusion in an upholstered room, and identifies correlations between demographic, clinical, and other factors with the length of the restriction periods.

## Materials and methods

This is a one-year retrospective evaluation study of restraint and seclusion in an upholstered room in a male acute, closed psychiatric ward in a government-owned mental health center, performed from January 1st, 2014 to December 31st, 2014. The data examined were from a period prior to recently issued guidelines on the use of restraint and seclusion by the Israeli Ministry of Health. The ward contains 50 beds and admits voluntary and involuntary patients. In this hospital there are only gender-separated acute closed wards, as is the case in most acute closed wards in Israel. The separated wards minimize eliminate interactions between male and female patients. The study population consisted of all ward admissions during the study period. The data were retrieved from each patient’s record. The analysis included the data on the following variables: age, marital status, education, race, ethnicity, psychiatric diagnosis according to ICD-10, length of hospital stay in days, type of event (seclusion or restraint), reasons for coercion, time of event, number of events per patient, total length of coercion in hours, number of previous hospitalizations, aggression in past and present treatment, and treatment during event. We evaluated correlations between the type of coercion (seclusion or restraint) and the number of staff members, their educational level, and the percent of inpatient occupancy in the ward at the time of event.

Seclusion is defined as placing the patient in a locked upholstered room. During seclusion, patients are observed by video monitoring, and there is communication via intercom. Mechanical restraint refers to the use of belts attached to a bed in a special single-bed room in order to restrict movement of each of the patient’s arms and legs. According to our internal hospital guidelines, mechanical restraint patients should be monitored continuously via a closed-circuit T.V. system and intermittently by the nursing staff who reassess the patient’s level of agitation and vital signs every half hour. During the use of coercive measures, patients receive either the regular treatment, but earlier than usual, or additional sedative drug therapy. There were two groups of additional sedative medications: 1) antipsychotics, and 2) benzodiazepines. Choice of the medication was based on the clinician’s judgment. The study was approved by the Institutional Review Board.

### Statistical analysis

The statistical analysis was performed in three phases. First phase: Descriptive statistics. In this phase we assessed the average and standard deviation for all the quantitative normally distributed variables and the median and range for all the quantitative non-normally distributed variables. We assessed percentages for the categorical variables. Second phase: univariate analysis. In this phase we compared the dependent variable with each of the independent categorical variables. For comparison of the dependent variable with the categorical variables, we used either the Mann-Whitney or the Kruskal-Wallis test, as appropriate. For a comparison of dependent variables with independent quantitative variables, we used Spearman’s rank correlation coefficient. Third phase: multivariable analysis. In this phase we used multivariate linear models. The regression model was constructed, including covariates that were found to be statistically significant in the univariate analysis and/or were clinically significant. Also, we checked possible interactions. All statistical tests and/or confidence intervals, as appropriate, were performed at α = 0.05 (2-sided). Statistical analysis was performed using SPSS version 21.

## Results

During 2014 there were 563 voluntary and involuntary hospitalizations in the study ward. Of these admissions, 166 (29.3%) were involuntary following a district psychiatrist’s decision, 115 (20.4%) were involuntary admissions ordered by a court for forensic observation and for psychiatric evaluation, 117 (20.8%) were involuntary admissions by court for forced treatment, and 165 (29.3%) were voluntary admissions. During this period, 176 (31.3% of all hospitalizations during 2014) patients were restricted and/or secluded. Since some patients underwent restraint or seclusion repeatedly, the total number of coercive interventions was 488: three hundred and eleven of them were restrained, and 177 secluded. Ninety eight percent of the patients had records of aggressive behavior. The mean duration of seclusion was 3.3 h (SD = 4.2), range 0.5–46.2 h; and the mean duration of mechanical restraint was 9.1 h (SD = 15.5), range 0.5–182.5 h. The mean age (SD) of subjects was 34.6 (11.9) years, range from 18 to 74 years. Demographic and clinical characteristic of these patients are presented in Table 2.

**Table 2:** Demographic and clinical characteristics of patients who underwent restraint or/and seclusion in an upholstered room (*N* = 176)

	Number	%
Origin		
Ashkenazic origin	69	39.2
Sephardic origin	62	35.2
Ethiopian	13	7.4
Muslim	19	10.8
Other	13	7.4
Marital status		
single	140	79.6
married	15	8.5
divorced	21	11.9
widowed	0	0.0
Age (years)		
18–20	8	4.5
21–30	77	43.8
31–40	37	21.0
41–50	37	21.0
51–60	12	6.8
61+	5	2.9
Education (years)		
0–5	1	0.6
6–8	17	9.6
9–12	92	52.3
13 +	20	11.3
No data	46	26.2
Diagnoses^a^		
Schizophrenia	97	52.8
Mental and behavioral disorders due to psychoactive substance use	33	17.9
Mood disorders	19	10.3
Examination for medico-legal reasons	21	11.4
Organic mental disorders	6	3.3
Personality disorders	5	2.7
Mental retardation	3	1.6
Cause for hospitalization:		
Forced hospitalization by regional psychiatrist	88	50.0
Forced psychiatric observation	50	28.5
Forced hospitalization by court	21	11.9
Voluntary agreement	17	9.6
Aggressive behavior in the past		
No	3	1.7
Yes	173	98.3

^a^Name of diagnoses according to ICD-10

Time spent in the upholstered room or being physically restrained was the longest in patients with personality disorders (median 4 h, range 1.5–182.5 h) compared with other diagnoses (*p* = 0.007), and the shortest time was among schizophrenia patients. The data are presented in Table 3.

**Table 3:** Diagnoses and length of coercive measures

Diagnosis	Number of coercive measures	Length of coercive measures in hours - median (minimum, maximum)	P*
Schizophrenia	238	3 (0.5, 60.0)	.007
Mental and behavior disorders due to psychoactive substance use	89	4 (0.5, 80.0)	
Mood disorders	56	4 (1.0, 31,5)	
Examination for medico-legal reasons	40	4 (0.75, 18.5)	
Organic mental disorders	44	3.5 (0.5, 29.5)	
Personality disorders	18	4 (1.5, 182.5)	
Mental retardation	5	4 (2.25, 7.0)

**Table 4:** Correlation between quantitative variables and length of coercive measures. The Kruskal-Wallis Test

Variables	Correlation Coefficient (hours)	P
Number of patients in ward	−0.10	0.830
Age of patients	−0.119	0.008
Origin of the patient		0.488
Years of education		0.433
Number of hospitalizations	−0.189	< 0.001
Duration of hospitalization	−0.255	< 0.001
Number of academic male nurses	−0.97	0.032
Number of academic female nurses	−0.128	0.004

A negative correlation was found between the length of coercion (in hours) and: age of patients (*r* = − 0.119, *p* = 0.001); number of previous hospitalizations (*r* = − 0.019, *p* < 0.001); duration of present hospitalization (*r* = − 0.255, p < 0.001); number of registered academic female nurses on duty (*r* = − 0.128, *p* < 0.005); and number of registered academic male nurses on duty (*r* = − 0.097, *p* < 0.05), (Table 4).

Table 5 presents the results of applying the multivariate linear regression for identifying factors associated with the duration of the coercive measures. There is a significant association between additional medication and duration of coercion.

**Table 5:** Multivariate linear regression model for identification of variables associated with the length of use of coercive measures

Variables	beta	p
Diagnoses		
Mood disorders	Ref.^a^	
Organic mental disorders	−.048	.307
Schizophrenia	−.143	.009
Mental and behavior disorders due to psychoactive	.018	.715
substance use		
Personality disorders	.073	.088
Mental retardation	−.038	.350
Family status		
Divorced	Ref.	
Single	−.107	.039
Married	−.050	.330
Sedative treatment during coercive measures		
Yes	Ref.	
No	−.023	.670
Antipsychotic treatment during coercive measures		
Yes	.188	.001
No	Ref.	
Reasons for coercive measures		
Violent towards oneself	.105	.041
Violent towards patients	.019	.728
Violent towards team	.037	.471
All above reasons	Ref.	
Coercive measures		
Seclusion	Ref.	
Restraint	.312	>.001
Degreed male nurses		
No	Ref.	
Yes	−.084	.038
Academic female nurses		
No	Ref.	
Yes	−.114	.005

^a^Ref. designates the referent element for each category examined

Patients who were restrained had a longer duration of coercion than patients who were secluded. In addition, patients who were divorced had a longer duration of coercion than those who were married.

Variables that were negatively associated with the length of coercion included: diagnosis of schizophrenia, single marital status, number of academic male nurses on duty, and the number of academic female nurses on duty. And, variables that were positively associated with the length of coercion included: lack of additional unscheduled medications during coercion, violence towards oneself as a cause of coercion, antipsychotic treatment during coercion, restraint as a coercive intervention.

## Discussion

Coercive measures are sometimes necessary to manage a patient with severely aggressive behavior. These measures can include physical force, mechanical devices, or drugs that temporarily restrict freedom of movement or control behavior. Although they are not a part of the patient’s standard treatment, according to the Joint Commission on Accreditation of Health Care Organizations (JCAHO) criteria [14], such intervention may be implemented as emergency treatment for patients with behavior that is dangerous to themselves or others.

Appropriate management by psychiatric ward staff of patients with disruptive behavior, creates a sense of security and can help find the balance between required therapeutic interventions and the need of preserving patients’ dignity. The indications for limitations of freedom in psychiatric facilities are not always well defined and may be prone to abuse. In many countries, there are no national guidelines regarding the use of coercive measures. The Israeli law for treatment of mentally ill patients (1991) sets a policy for using means of coercion, as follows:

A. restraint method may refer as to seclusion or restriction
B. use of coercive measures for the hospitalized patients should be made only to the extent required for medical treatment of the patient or to prevent danger to himself or others
C. the medical directive regarding the use of a coercive measure should be given in writing by a physician for a limited period, in a state of emergency and in the absence of a physician the nurse is permitted to provide a coercive instruction.

Published studies performed in different countries indicate that coercive measures are used in 100% of the wards in Germany, 60% of those in Switzerland, and, for all practical purposes, in none of the wards in Great Britain where physical restraint is applied only along with pharmacological restraint and for a very short period of time (mean 12 min) [4]. Such a difference can be explained by cultural and demographic characteristics between patients of different countries, number of personnel per patient, overcrowding and physical conditions of the facilities.

Our findings from this retrospective study demonstrate that during the study period about one third of all admitted patients (31.3%) required the use of coercive measures. These results correspond to other studies performed on a similar sample size with similar duration [11, 15]. In our study, since most of the patients needing coercive measures were dangerous for themselves, more were restrained than secluded. It is hard to prevent self-harm without restraints.

Our findings concerning age, number and duration of hospitalizations are similar to those of other studies. McLaughlin et al. found that all coercive measures were associated with patients staying longer in hospital [16]. Caqueo-Urizar et al. [17] noted that younger age is associated with more violent behavior, since young adulthood is a critical period at risk for aggressive behavior. According to the study performed by Dumais et al., younger age and a longer stay in hospital are predictors of an episode of seclusion with restraint [18].

Although patients with a principal diagnosis of a personality disorder were least likely to undergo coercive measures in our sample (about 3%), these patients were restricted for the longest period in comparison with patients with other diagnoses. Use of physical restraint in this particular group brought temporary relief from the feelings of regret and remorse about the trouble they had caused others during their repeated cycles of violence and aggression [19]. In our opinion, the explanation for this discrepancy may be related to the fact that personality disorder patients, especially those with aggressive and violent behavior, frequently cause a negative countertransference in the personal staff. In some cases, hopefully rare, staff might use these measures as punishment. We suggest that this unconscious reaction may lead to a longer time of restriction. Therefore it is important to educate staff and raise their awareness about this issue. In addition, physical restraint is often not the sole solution for patients with a personality disorder who exhibit aggressive and violent behavior. They also need pharmacological and psychological treatment in order to achieve long-term, effective management of their behavior.

Conversely, patients with schizophrenia (the most prevalent diagnosis - about 53%) were under coercive measures for the shortest time in comparison to patients with other diagnoses. A similar trend was observed in studies performed by Dumais et al. [20], Caqueo-Urizar [17], and Huber et al. [21]. According to Beck et al. [10], schizophrenia or other psychoses appear to be a risk factor for a single episode of restriction, but not for multiple episodes. The review by Beghi et al. [3] notes that in some studies a diagnosis of schizophrenia may increase the risk of aggressiveness and restraint [3].

Interestingly, we have found that female nurses generally used less restraint than males, but both academic male and female nurses used less coercive measures. Similar data were described in some studies, which found that a male staff member is more likely to use restraint that a female staff member [22, 23]. These researchers believe that aggressiveness tends to be directed against people of the same gender and, given that more male patients are restrained, this is more likely to be done by male staff. Our results emphasize that the level of academic education of the staff is fundamental and influences the duration of coercion. Our opinion is that the key principles and initial management of the agitated patient should be part of the syllabus of nursing students.

Other studies have also shown that variables, such as young age [10, 20, 24, 25], previous hospital admissions [25], length of hospital stay [10, 20, 25, 26], involuntary status of the admission [10, 27], diagnosis of bipolar disorder [20] and personality disorder [10, 18, 20, 21], are associated with restraint of patients.

Some authors have found that inpatient overcrowding is associated with an increased rate of aggressive behavior [17, 18, 21, 28, 29]. In contrast, we did not find any association between the degree of crowding in the ward and frequency or duration of coercive measures.

As others [10, 11, 28, 29], we also did not find an association between educational level, ethnic origin of patients and frequency or length of coercive measures. However, some studies have found associations between low educational level and ethnicity and higher aggression level and longer duration of restraint [15, 17, 30, 31].

The main limitation of our study is that it was a retrospective one. Thus we cannot be certain that the associations we have demonstrated are causal.

It should be noted that in the last 2 years there is a public debate in Israel concerning the issue of patients’ restraint in psychiatric units. The practice of restraint has been dramatically diminished following this debate. Future studies should be designed to evaluate the results of these changes, for example, whether they have had an influence on patients’ violence against staff and other patients.

## Conclusions

The use of physical restraint should be based on the prevailing needs of the patient and should be used only as a “last resort” [19]. According to Human Rights Working Group, seclusion is “the restriction of a person’s freedom, without his or her consent, by locking him or her in a room. It can be justified only on the basis of a clearly identified and significant risk of serious harm to others that cannot be managed with greater safety by any other means” [32].

Locking someone alone in a room is a serious intervention and must be carefully regulated and monitored, in order to avoid abuse. We believe that it can be done, when essential, by applying a rigorous set of principles for its use and ensuring that there is a careful framework for monitoring the practice at the local level. The use of seclusion, while less restrictive, should always and only be applied for the benefit of the patient. Even so, the use of restriction can cause distress and psychological harm and can increase the potential risk of self-harm [19]. Therefore, evaluation of the intended benefit should be carried out after each episode of restraint.

We assume that three factors associated with length of coercion time should be taken in account: 1) personality disorder may lengthen it; 2) presence, on duty, of male and female nurses with academic degrees can reduce length of coercion; 3) giving sedative medication during coercive measures also could diminish length of restriction.

The application of coercive measures should be used as an adjunct to pharmacological treatment and psychological support, to achieve long-term, effective management of the patients’ disturbances. Furthermore, staff should have access to routine supervision with regard to their practice. To prevent the risks associated with the use of restraint in psychiatry, it is necessary to train staff with courses that encourage the use of diverse methods of managing aggressive patients (de-escalation techniques) [33–35].

We believe that restraint use should be minimized, but is sometimes necessary to prevent and manage violent, aggressive, and self-harming behavior of patients. It will be used most fairly and effectively when it results from a decision-making process by highly skilled staff based on careful observation and is then is carefully monitored.
